# 1021. Utility of Cell-Free DNA Sequencing in Diagnosing *Murine typhus* in Children

**DOI:** 10.1093/ofid/ofab466.1215

**Published:** 2021-12-04

**Authors:** Victoria M Medina-Carbonell, Enrique A Yespica-Otaola, Kim Beatty, Jaime Fergie

**Affiliations:** 1 Universidad Central de Venezuela, Corpus Christi, Texas; 2 Universidad Central de Venezuela, Corpus Chrsiti, Texas; 3 Driscoll Children’s Hospital, Corpus Christi, Texas; 4 Infectious Disease, Driscoll Children’s Hospital, Corpus Christi, Texas

## Abstract

**Background:**

Murine typhus is a zoonotic infection caused by Rickettsia typhi and transmitted through infected fleas. Geographic distribution within the United States is limited primarily to South Texas and Southern California. Infection is typically associated with a triad of fever, headache, and rash, although is only present in one-third of cases. Immunofluorescence assay (IFA) is currently the gold standard for diagnosis, but it has its limitations as it is dependent on the time to seroconversion and has low specificity due to cross-reactivity among other rickettsial species. Cell-free DNA (cfDNA) sequencing for broad-range pathogen detection may offer higher sensitivity at the early stages of the disease.

**Methods:**

We performed a retrospective electronic medical record search of children with cfDNA sequencing detection of Murine typhus hospitalized at Driscoll Children’s Hospital, Corpus Christi, Texas, between June 2020 and May 2021.

**Results:**

We found 4 children (range 9-15 year-old) positive for R. typhi by cfDNA sequencing. All patients presented with fever of unknown origin and rash. Also, 2 patients were diagnosed with pneumonia. One patient exhibited severe illness with acute kidney injury, elevation of transaminases and encephalitis that warranted admission to the pediatric intensive care unit. All patients defervesced and improved within 48 hours of doxycycline initiation; average length of stay 6 days (range 3-12 days). In one patient, M. typhus was detected by Karius® test only, in the other three was concordant with serology.

**Conclusion:**

We highlight next-generation cfDNA sequencing as a useful tool in identifying the etiologic agent of patients with fever of known origin, where murine typhus is one of the possible etiologies. Preventing extensive laboratory workup and subsequent delay of assessment and management. The rapid turnaround time of cfDNA test allows for de-escalation of therapy and initiation of appropriate treatment.

**Disclosures:**

**Jaime Fergie, MD**, **AstraZeneca** (Scientific Research Study Investigator)**Explify** (Speaker’s Bureau)**Karius** (Speaker’s Bureau)**Pfizer, Merck, AstraZeneca, and Sanofi** (Speaker’s Bureau)**Pfizer, Merck, Sanofi, and Moderna** (Consultant, Advisor or Review Panel member)

